# Feasibility and validity of accelerometer measurements to assess physical activity in toddlers

**DOI:** 10.1186/1479-5868-8-67

**Published:** 2011-06-26

**Authors:** Eveline Van Cauwenberghe, Jessica Gubbels, Ilse De Bourdeaudhuij, Greet Cardon

**Affiliations:** 1 Department of Movement and Sport Sciences, University of Ghent, Watersportlaan 2, 9000 Ghent, Belgium; 2 Department of Health Promotion, Maastricht University Medical Centre+, PO Box 616, 6200 MD Maastricht, The Netherlands

**Keywords:** ActiGraph, Observational System for Recording Physical Activity in Children-Preschool (OSRAC-P), Feasibility, Criterion validity, Predictive validity, Accelerometer cut points, Toddlers

## Abstract

**Background:**

Accelerometers are considered to be the most promising tool for measuring physical activity (PA) in free-living young children. So far, no studies have examined the feasibility and validity of accelerometer measurements in children under 3 years of age. Therefore, the purpose of the present study was to examine the feasibility and validity of accelerometer measurements in toddlers (1- to 3-year olds).

**Methods:**

Forty-seven toddlers (25 boys; 20 ± 4 months) wore a GT1M ActiGraph accelerometer for 6 consecutive days and parental perceptions of the acceptability of wearing the monitor were assessed to examine feasibility. To investigate the validity of the ActiGraph and the predictive validity of three ActiGraph cut points, accelerometer measurements of 31 toddlers (17 boys; 20 ± 4 months) during free play at child care were compared to directly observed PA, using the Observational System for Recording Physical Activity in Children-Preschool (OSRAC-P). Validity was assessed using Pearson and Spearman correlations and predictive validity using area under the Receiver Operating Characteristic curve (ROC-AUC).

**Results:**

The feasibility examination indicated that accelerometer measurements of 30 toddlers (63.8%) could be included with a mean registration time of 564 ± 62 min during weekdays and 595 ± 83 min during weekend days. According to the parental reports, 83% perceived wearing the accelerometer as 'not unpleasant and not pleasant' and none as 'unpleasant'. The validity evaluation showed that mean ActiGraph activity counts were significantly and positively associated with mean OSRAC-P activity intensity (r = 0.66; p < 0.001; n = 31). Further, the correlation among the ActiGraph activity counts and the OSRAC-P activity intensity level during each observation interval was significantly positive (ρ = 0.52; p < 0.001; n = 4218). Finally, the three sedentary cut points exhibited poor to fair classification accuracy (ROC-AUC: 0.56 to 0.71) while the three light PA (ROC-AUC: 0.51 to 0.62) and the three moderate-to-vigorous PA cut points (ROC-AUC: 0.53 to 0.57) demonstrated poor classification accuracy with respect to detecting sedentary behavior, light PA and moderate-to-vigorous PA, respectively.

**Conclusions:**

The present findings suggest that ActiGraph accelerometer measurements are feasible and valid for quantifying PA in toddlers. However, further research is needed to accurately identify PA intensities in toddlers using accelerometry.

## Background

Early childhood (defined as between 0 and 5 years of age) is one of the critical developmental periods in life in which health behaviors such as physical activity are established [1,2]. Regular physical activity during early childhood protects against unhealthy weight gain and contributes to the overall development and well-being of children under 5 years of age [3,4]. In line with this acknowledgement, governments and professional groups have developed physical activity recommendations for children from birth to age 5 in accordance with their developmental stage, namely for infants (0- to 1-year olds), toddlers (1- to 3-year olds) and preschoolers (3- to 5-year olds) [5,6].

One of the challenges faced by researchers trying to promote physical activity is having access to accurate and practical instruments to measure physical activity [7-9]. Valid measurement of physical activity in children under 5 years of age is challenging, largely due to the sporadic and intermittent nature of their activity behavior [7-9]. Proxy reports from parents can be useful for rank ordering children across the 0 to 5 age group on physical activity behavior. However, inaccurate estimates of the amount and intensity of physical activity remain a primary concern with this approach [8,10]. On the other hand, accelerometers and direct observation have become well-established and preferred methods for monitoring physical activity performed by children aged 0 to 5 years [7,8]. Both accelerometers and direct observation are developmentally appropriate and allow detection of short spurts of activity [8,11]. Accelerometers have the additional advantage of enabling objective quantification of the frequency, intensity and duration of physical activity during all waking hours over several days. Moreover, the relatively low researcher and participant burden associated with accelerometers, and their lower cost as compared with direct observation makes them particularly attractive [7-10]. Therefore, accelerometers are currently considered to be the most promising tool for measuring habitual physical activity in free-living children aged 0 to 5 years [7,8,10].

Nevertheless, validity, reliability and feasibility of accelerometer measurements have only been established in preschoolers [10,11]. Further, accelerometers have never been applied to evaluate physical activity levels in children younger than 3 years [12]. Only two studies could be located describing physical activity levels in toddlers, one using direct observation [13] and one using parental reports [14]. More research in these areas is warranted to gain a comprehensive understanding of physical activity during the infant and toddler years [10,12]. Additionally, testing accelerometer feasibility and validity and describing physical activity levels in infants, toddlers and preschoolers separately are a necessity as movement patterns during these unique developmental periods differ, and physical activity recommendations are specific for each age group [3,5,6,10]. During the first 12 months of life, activity is characterized by the learning of rudimentary movement skills, such as rolling, reaching, grasping, sitting, crawling, standing and walking. With the onset of walking, the toddler period begins and children start to develop proficiency in locomotor skills such as running, jumping, hopping, galloping and skipping. Further, manipulative and stability skills also begin to emerge in this period. Finally, the preschool period generally encompasses ages 3 to 5 and is characterized by further development and refinement of locomotor, manipulative and stability skills. Next to these differences in activity patterns, daytime naps are more common in children younger than 3 years of age compared to older children suggesting that younger children have less time during the day to be physically active and that the number of hours of monitoring required to represent a typical day might be less in this age group [10].

To date, the potential of accelerometers to assess physical activity in children under 3 years of age is largely unknown. Accordingly, the first purpose of the present study was to test the feasibility of ActiGraph accelerometer-based physical activity assessments in toddlers. A second purpose was to examine the validity of the ActiGraph accelerometer in toddlers. Finally, as toddler specific accelerometer cut points are currently lacking, the predictive validity of three independently developed ActiGraph cut points for classifying physical activity intensity among preschoolers was evaluated in toddlers.

## Methods

### Participants

Forty-four child care centers from Ghent, Flanders, Belgium were randomly selected from the official database of the Flemish governmental agency "Child & Family" (Kind & Gezin) [15]. The head of each child care center was contacted by phone and 27 (61.4%) were willing to participate. Following approval from the head of each center, all parents of 1- to 2-year old children were invited to enroll their child in the present study (n = 272) using written information letters and consent forms, distributed directly to parents by the child care center staff. The study was approved by the Ethics Committee of the University Hospital of Ghent. Fifty-five parents from 11 child care centers agreed to let their child participate in the present study (20.2% of eligible children). Children were finally included in the study if it was observed by a researcher that the child could walk independently. Eight children did not meet this criterion and were consequently excluded, resulting in a final sample of 47 toddlers from 11 child care centers. Of the 47 participants, 31 (63.3%) were observed during free play. Descriptive characteristics for all participants and the observed participants are presented in Table 1.

**Table 1:** Participant characteristics

Characteristic	Total sample (n = 47)	Observed sample (n = 31)	t or chi square value	p-value
Age (months)	20 ± 4	20 ± 4	t = -0.353	0.73

Age distribution (%)			χ^2 ^= 0.368	0.83
12-17 months	34.0	35.5		
18-23 months	41.1	45.2		
24-30 months	14.9	19.3		

Male (%)	53.2	54.8	χ^2 ^= 0.02	0.89

Height (cm)	79.5 ± 5.0	79.0 ± 5.0	t = 0.442	0.66

Body mass (kg)	11.2 ± 1.4	11.2 ± 1.5	t = -0.152	0.88

BMI (kg/m^2^)	17.7 ± 1.5	18.0 ± 1.4	t = -0.805	0.42

BMI z-score	1.2 ± 1.0	1.4 ± 0.9	t = -0.850	0.40

### Procedure

All participants wore an accelerometer during waking hours for 6 consecutive days, including 4 weekdays and 2 weekend days. Parents and child care staff were instructed to only remove the accelerometer when the toddlers performed water-based activities (e.g., bathing, swimming) and during sleeping and napping because the aim was to define physical activity during waking time. During the measurement period, no reminders were provided to the parents or the child care staff to comply with the protocol. A diary was provided for the parents to log the times the accelerometer was put on and taken off and the reason for doing so. Additionally, parents reported their child's average sleeping and napping time during weekdays and weekend days. Finally, parents were asked to report on toddler's perception of wearing the accelerometer on a 5-point scale with endpoints ranging from 1 (very unpleasant) to 5 (very pleasant). Any other remarks about wearing the accelerometer were also recorded. On the first day of the protocol, the accelerometer was attached and toddler's height and weight were measured by a researcher. After the accelerometer was attached, toddler's activity behavior was videotaped by a researcher during free play at child care (indoors or outdoors) using a Sony Digital Handycam DCR-PC101E. A digital watch was synchronized with the computer used to upload data and download data from the accelerometers. The start and stop times of toddler's free play were recorded. On day 6, the accelerometer and the diary were collected by a researcher at the child care center.

## Measures

### Child characteristics

While toddlers were barefoot and in light clothing, height was measured to the nearest 0.1 cm using a portable stadiometer SECA 214. Weight was measured to the nearest 0.1 kg using a digital scale SECA 813. Height and weight were used to determine the body mass index (BMI; kg/m^2^). BMI z-scores were calculated on the basis of the WHO reference data using the LMS method [16]. A z-score indicates by how many standard deviations a child deviates from the age and sex specific reference value. Toddlers' demographics (gender and date of birth) were acquired through the child care centers.

### GT1M ActiGraph accelerometer

Toddlers' physical activity levels were objectively measured using the GT1M ActiGraph accelerometer, a uniaxial accelerometer designed to detect vertical accelerations. At present, ActiGraph accelerometers are the most widely used accelerometers for physical activity research in children and adolescents [9,11,17]. Consistent with previous research in preschoolers, the accelerometers were fastened snugly around the waist and positioned on the right hip, using an adjustable elastic belt [10,17,18]. Accelerometers were programmed to start measuring on the first day and to record data every 15 seconds [9,10,19]. After data collection, accelerometers were downloaded for subsequent data reduction and analysis. The software Meterplus 4.2 was used to screen and clean the accelerometer data from the 6 days of measurement [20]. Because of the absence of methodological studies investigating accelerometer data reduction in toddlers, decision rules in accordance with previous research in preschoolers were applied. Both the first and the last day of the registration period were omitted because these days were incomplete [21]. Periods containing 10 minutes or more of consecutive zero activity counts were regarded as non-wearing time and were excluded [10,21]. The minimum number of minutes with recorded accelerometer data (registration time) required to constitute an eligible weekday and weekend day was determined by defining the period during which at least 70% of the study population had recorded accelerometer data and 80% of that observed period was the minimum registration time [18,21]. Days on which participants did not achieve the minimum registration time were considered as non-eligible days and were excluded. Because of toddlers' sleeping and napping patterns, it was expected that toddlers' registration time would be lower compared to preschoolers' registration time. Therefore, the 70/80 rule was preferable to a priori determined criteria previously used in preschoolers (e.g., a minimum registration time of 8 hours) as this rule uses the sample from the study under investigation [18]. Minimum registration time was defined for weekdays and weekend days separately as this may potentially vary [10,18,19]. Ultimately, participants were included in the analyses if data were available for 3 valid days [10,22]. As a measure of toddlers' total physical activity, mean counts per 15 seconds during weekdays and weekend days was calculated.

To compare the accelerometer data with physical activity levels during the free play session as measured using direct observation, the start and stop times were applied to extract the corresponding accelerometer data, namely activity counts per 15 seconds epoch. Each 15 seconds epoch was subsequently classified as sedentary behavior, light physical activity and moderate-to-vigorous physical activity. ActiGraph cut points to define physical activity intensities have not yet been developed in toddlers and, therefore, cut points specifically developed for the age group nearest to toddlers (3- to 5-year olds) using a 15 seconds measurement interval were selected. The cut points developed in the following calibration studies were used: Pate et al. [23,24] for 3- to 5-year old children (sedentary behavior: ≤ 37; light physical activity: 38 - 419; moderate-to-vigorous physical activity: ≥ 420), Sirard et al. [25] for 3-year old children (sedentary behavior: ≤ 301; light physical activity: 302 - 614; moderate-to-vigorous physical activity: ≥ 615) and Van Cauwenberghe et al. [21] for 5-year old children (sedentary behavior: ≤ 372; light physical activity: 373 - 584; moderate-to-vigorous physical activity: ≥ 585).

### Observational System for Recording Physical Activity in Children-Preschool

The criterion measure of physical activity intensity during the free play sessions was assessed by means of an adapted version of the Observational System for Recording Physical Activity in Children-Preschool (OSRAC-P) [26]. OSRAC-P is a focal child, momentary time sampling observation system and scores children's physical activity intensity level every 30 seconds on a 1 to 5 scale where 1 = stationary and motionless; 2 = stationary with movement of limbs or trunk; 3 = slow, easy movement; 4 = moderate movement; and 5 = fast movement [27]. As such, the original coding protocol does not allow for frequent recording of physical activity, possibly resulting in an inability to adequately assess the typical sporadic and intermittent physical activity patterns of young children [7]. In addition, the 30 seconds observation interval does not correspond with the chosen accelerometer measurement interval of 15 seconds. Consequently, it was decided to use a computerized 15 seconds-by-15 seconds coding protocol using the software Vitessa 0.1 [28]. The free play session was videotaped and afterwards the video footage was downloaded and scored every 15 seconds, except for instances when the toddler was not visible in the video footage (e.g., behind furniture, other child or adult). In accordance to the manual and to previous research in toddlers, the highest level of intensity achieved by the toddler during each 15 seconds interval was recorded [13,27]. Afterwards, intervals coded as stationary and motionless or stationary with movement of limbs or trunk were classified as sedentary behavior, intervals coded as slow, easy movement as light physical activity and intervals coded as moderate or fast movement as moderate-to-vigorous physical activity [27]. In addition, the OSRAC-P scale assessing toddler's topography of physical activity behavior (e.g., running, sitting, walking, riding) was used to score toddler's physical activity type during each observation interval.

Before data collection, two observers were engaged in a training protocol for 40 hours, including reading OSRAC-P articles, studying observation procedures and practicing and discussing coding definitions. To assess inter-observer reliability, it is recommended that approximately 12% of observations should be independently coded [27]. Inter-observer agreement scores, using stringent interval-by-interval comparisons, were calculated separately for toddler's physical activity level and physical activity type. The remaining observations were coded by both observers individually.

### Statistical analyses

To investigate the feasibility of the ActiGraph accelerometer in toddlers, descriptive statistics of the feasibility variables were conducted. Paired Samples t-tests were performed to check for differences in registration time and daily activity level between weekdays and weekend days and Independent Samples t-tests and crosstabs to examine demographic or anthropometric differences between the total sample and the observed sample and between toddlers providing eligible and non-eligible accelerometer data. To evaluate the criterion validity of the ActiGraph accelerometer against direct observation, the correlation between mean accelerometer activity counts and mean directly observed activity intensity levels during the observation of each toddler was calculated. Further, the correlation between the accelerometer activity counts and the directly observed activity intensity during each observation interval across all observations was calculated. Depending on the normal distribution of the variables, Pearson (skewness < 0.7) or Spearman (skewness ≥ 0.7) correlations were performed. For all analyses, SPSS for Windows 15.00 was used and statistical significance was set at an alpha level of 0.05. The predictive validity of each accelerometer cut point was tested by performing two different analyses using Medcalc 11.4.4. First, Bland-Altman plots were conducted to determine systematic bias and 95% limits of agreement between observed and predicted time in each activity intensity by each cut point [29]. Second, the ability to accurately classify sedentary behavior, light physical activity and moderate-to-vigorous physical activity was evaluated for each set of cut points by calculating sensitivity, specificity and area under the Receiver Operating Characteristic curve (ROC-AUC). ROC-AUC provides a measure of classification accuracy that jointly considers sensitivity and specificity [30]. A Receiver Operating Characteristic curve plots the false positive rate (1 - specificity) on the x-axis and the true positive rate (sensitivity) on the y-axis. ROC-AUC of 1 represents perfect classification, whereas an area of 0.5 represents a complete absence of classification accuracy. ROC-AUC values of ≥ 0.90 are considered excellent, 0.80 - 0.90 good, 0.70 - 0.80 fair and < 0.70 poor [31].

## Results

### Feasibility

During weekdays and weekend days, a minimum registration time of 452 min (7.5 h) and 464 min (7.7 h) per day, respectively, was determined using the 70/80 rule. Twenty-nine weekdays and 36 weekend days did not meet the minimum registration time and were excluded. This resulted in three toddlers having 0 eligible days, six having 1 eligible day, eight having 2 eligible days, 19 having 3 eligible days and 11 having 4 eligible days. Ultimately, accelerometer measurements of 30 toddlers (63.8%) were included. No demographic or anthropometric differences were observed between toddlers providing eligible or non-eligible accelerometer data (all p > 0.05). The mean registration time of the included toddlers was 564 ± 62 min (9.4 h; range: 452 - 714 min) during weekdays and 595 ± 83 min (9.9 h; range: 468 - 846 min) during weekend days. The difference in registration time between weekdays and weekend days was not statistically significant (t = -1.609; p = 0.115). Daily activity level was 126 ± 39 counts/15 s (range: 51 - 213 counts/15 s) during weekdays and 115 ± 35 counts/15 s (range: 62 - 209 counts/15 s) during weekend days and did not differ significantly (t = 1.526; p = 0.135). The diary was filled out by 39 parents (83.0%) and all logged the times when the accelerometer was taken off for sleeping, napping, bathing, swimming and when the accelerometer was put back on. Six parents (15.4%) reported that they forgot to put on the accelerometer during one or more days; five of these children (83.3%) provided non-eligible accelerometer data. Five parents (12.8%) reported that there was some delay in refitting the monitor after their toddler had woken up or taken a nap during one or more days; one of these children (20.0%) provided non-eligible accelerometer data. During weekdays, median sleeping and napping time was 11.3 h (IQR: 10.9 - 11.8) and 2.0 h (IQR: 1.9 - 3.0), respectively, and during weekend days 11.0 h (IQR: 10.9 - 12.0) and 2.0 h (IQR: 2.0 - 3.0), respectively. Almost all the parents (82.9%) reported that their toddler found it 'not unpleasant and not pleasant' to wear the accelerometer while none found it 'very unpleasant' or 'unpleasant' to wear the accelerometer (median: 3; IQR: 3 - 3). Three parents reported that sometimes the accelerometer did not stay on the correct position and one parent informed that this was due to the curiosity of his/her child.

### Free play observations

Sixteen of the 47 toddlers could not be observed because they were having dinner, were taking a nap or were being picked up by their parents to go home. Table 1 shows that no significant demographic or anthropometric differences were found between the observed sample and the total sample (all p > 0.05). Seventeen (54.8%) free play observations were indoors, 10 (32.3%) were outdoors and four (12.9%) were both indoors and outdoors. The observation period ranged from 19.5 (78 observation intervals) to 60.0 min (240 observation intervals) resulting in a total of 4553 observation intervals of 15 seconds. Of these observation intervals, 335 (7.4%) could not be scored because the child was not visible. As a result, 4218 observation intervals could be used. To assess inter-observer agreement, four randomly selected participants were independently coded by two observers (490 observation intervals; 11.6%). Inter-observer agreement was 91% and 96% for toddler's physical activity level and type, respectively.

Tables 2 and 3 display descriptive statistics for OSRAC-P activity intensity level (scale 1 - 5) and ActiGraph activity counts during each intensity level and activity type. The mean intensity level across all the observations, as assessed by OSRAC-P and accelerometry, was 2.6 ± 0.9 (inter-child range: 1.8 - 3.3) and 137 ± 199 activity counts/15 s (inter-child range: 30 - 291 counts/15 s), respectively. Toddlers spent the majority of the observation intervals in stationary and motionless to stationary with movement of limbs or trunk behavior (50.4%) and were observed in slow, easy movement (36.3%) and moderate to fast movement (13.3%) less frequently. The inter-child range for physical activity level was 14.8% - 81.2% for stationary and motionless to stationary with movement of limbs or trunk behavior, 17.1% - 64.9% for slow, easy movement and 0.9% - 38.5% for moderate to fast movement. Three physical activity types, namely sit and squat (24.3%), stand (24.3%) and walk (33.1%), accounted for the greatest proportion of toddler's physical activity topography during the free play observations.

**Table 2:** ActiGraph activity counts during each OSRAC-P activity intensity level

OSRAC-P activity intensity level	ActiGraph activity counts/15 s	
	
	Median	IQR
Stationary and motionless to stationary with movement of limbs or trunk behavior (sedentary behavior; n = 2125 observation intervals)	10	0 - 69

Slow, easy movement (light physical activity; n = 1531 observation intervals)	100	29 - 230

Moderate to fast movement (moderate-to-vigorous physical activity; n = 562 observation intervals)	229	104 - 385

OSRAC-P, Observational System for Recording Physical Activity in Children-Preschool; IQR, interquartile range

**Table 3:** OSRAC-P activity intensity level and ActiGraph activity counts during each OSRAC-P activity type

OSRAC-P activity type	OSRAC-P activity intensity level		ActiGraph activity counts/15 s	
	
	Median	IQR	Median	IQR
Stand (n = 1025 observation intervals)	2	2 - 2	10	0 - 46

Sit/squat (n = 1024 observation intervals)	2	2 - 2	9	0 - 96

Lie down (n = 48 observation intervals)	2	2 - 2	120	16 - 387

Dance (n = 10 observation intervals)	2	2 - 2	2	0 - 6

Rock (n = 3 observation intervals)	2	2 - 2	100	50 - 214

Walk (n = 1398 observation intervals)	3	3 - 3	97	31 - 222

Ride/peddle (n = 133 observation intervals)	3	3 - 3	77	0 - 230

Pull/push (n = 131 observation intervals)	3	3 - 4	176	54 - 346

Crawl (n = 78 observation intervals)	3	3 - 3	288	73 - 473

Climb (n = 48 observation intervals)	3	3 - 3	351	192 - 503

Roll (n = 4 observation intervals)	3	2 - 3	162	41 - 387

Rough & tumble (n = 1 observation interval)	3	3 - 3	3	3 - 3

Throw (n = 156 observation intervals)	4	3 - 4	246	128 - 404

Jump/skip (n = 15 observation intervals)	4	3 - 4	310	205 - 498

Run (n = 144 observation intervals)	5	4 - 5	259	140 - 371

OSRAC-P, Observational System for Recording Physical Activity in Children-Preschool; IQR, interquartile range

### Criterion validity

Mean ActiGraph activity counts were significantly and positively associated with mean OSRAC-P activity intensity levels (r = 0.66; p < 0.001; n = 31). Further, the correlation among the ActiGraph activity counts and the OSRAC-P activity intensity level during each observation interval across all observations was also significant (ρ = 0.52; p < 0.001; n = 4218).

### Predictive validity

Bland-Altman analyses for each set of cut points are displayed in Table 4. The Pate sedentary behavior cut point tended to underestimate stationary and motionless to stationary with movement of limbs or trunk behavior (mean difference of 1.5 min) while the Sirard and Van Cauwenberghe sedentary behavior cut points tended to overestimate stationary and motionless to stationary with movement of limbs or trunk behavior (mean difference of -11.5 min and -13.0 min, respectively). The smallest bias was achieved when the Pate sedentary behavior cut point was used. Slow, easy movement was underestimated when the Sirard and Van Cauwenberghe light physical activity cut points (mean difference of 8.2 min and 9.9 min, respectively) were applied while slow, easy movement was overestimated with the Pate light physical activity cut point (mean difference of -3.0 min). Using the Pate cut point resulted in the smallest bias in predicted slow, easy movement. All three cut points underestimated moderate to fast movement (mean difference of 1.5 to 3.3 min) with the Pate moderate-to-vigorous physical activity cut point demonstrating the highest level of agreement.

**Table 4:** Mean difference between observed and predicted time in each activity intensity

OSRAC-P activity intensity level	Observed time (mean min ± SD)	Cut point	Mean difference (min)	ULOA (min)	LLOA (min)
Stationary and motionless to stationary with movement of limbs or trunk behavior (sedentary behavior)	68.5 ± 37.6	Pate (≤ 37)	1.5	14.0	-11.0
		
		Sirard 3-year olds (≤ 301)	-11.5	2.1	-25.0
		
		Van Cauwenberghe (≤ 372)	-13.0	0.6	-26.9

Slow, easy movement (light physical activity)	49.4 ± 21.2	Pate (38 - 419)	-3.0	8.2	-14.2
		
		Sirard 3-year olds (302 - 614)	8.2	18.6	-2.2
		
		Van Cauwenberghe (373 - 584)	9.9	19.8	-0.1

Moderate to fast movement (moderate-to-vigorous physical activity)	18.1 ± 16.3	Pate (≥ 420)	1.5	8.2	-5.2
		
		Sirard 3-year olds (≥ 615)	3.3	0.5	-3.9
		
		Van Cauwenberghe (≥ 585)	3.2	10.4	-4.1

OSRAC-P, Observational System for Recording Physical Activity in Children-Preschool; ULOA, upper limits of agreement; LLOA, lower limits of agreement

In Table 5 overall agreement, sensitivity, specificity and ROC-AUC values for each cut point to categorize activity counts as stationary and motionless to stationary with movement of limbs or trunk behavior, slow, easy movement and moderate to fast movement are presented. The Pate cut points showed the highest level of agreement (58.3%) across all intensity levels. The Pate sedentary behavior cut point exhibited reasonable levels of sensitivity (67.0%) and specificity (75.4%), resulting in fair classification accuracy (ROC-AUC: 0.71). The Sirard and Van Cauwenberghe sedentary behavior cut points were highly sensitive (91.8% and 94.4%, respectively) but not specific (23.9% and 17.2%, respectively) and consequently classification accuracy was poor (ROC-AUC: 0.58 and 0.56, respectively). For slow, easy movement, classification accuracy for all three light physical activity cut points was poor (ROC-AUC: 0.51 to 0.62). For the Pate cut point, this was a function of low sensitivity (60.0%) and specificity (63.2%). Poor performance of the Sirard and Van Cauwenberghe cut points was the result of poor sensitivity (14.6% and 9.0% respectively). For moderate to fast movement, all moderate-to-vigorous physical activity cut points demonstrated poor classification accuracy (ROC-AUC: 0.53 to 0.57) and this resulted primarily from low sensitivity (8.9% to 21.5%).

**Table 5:** Agreement, sensitivity, specificity and area under the Receiver Operating Characteristic curve for each cut point

Cut point		Pate		Sirard 3-year olds		Van Cauwenberghe	
Overall agreement%		58.3		52.7		52.2	

OSRAC-P activity intensity level	Test		95% CI		95% CI		95% CI

Stationary and motionless to stationary with movement of limbs or trunk behavior (sedentary behavior; n = 2125 observation intervals)	Sensitivity%	67.0	64.9 - 68.9	91.8	90.5 - 92.9	94.4	93.3 - 95.3
	
	Specificity%	75.4	73.5 - 77.2	23.9	22.0 - 25.7	17.2	15.6 - 18.8
	
	ROC-AUC	0.71	0.70 - 0.73	0.58	0.56 - 0.59	0.56	0.54 - 0.57

Slow, easy movement (light physical activity; n = 1531 observation intervals)	Sensitivity%	60.0	57.5 - 62.4	14.6	12.8 - 16.4	9.0	7.6 - 10.6
	
	Specificity%	63.2	61.3 - 65.0	89.0	87.8 - 90.2	93.7	92.7 - 94.6
	
	ROC-AUC	0.62	0.60 - 0.63	0.52	0.50 - 0.53	0.51	0.50 - 0.53

Moderate to fast movement (moderate-to-vigorous physical activity; n = 562 observation intervals)	Sensitivity%	21.5	18.2 - 25.2	8.9	6.7 - 11.6	10.0	7.6 - 12.7
	
	Specificity%	93.1	92.2 - 93.9	97.1	96.5 - 97.6	96.9	96.2 - 97.4
	
	ROC-AUC	0.57	0.56 - 0.59	0.53	0.52 - 0.55	0.53	0.52 - 0.55

OSRAC-P, Observational System for Recording Physical Activity in Children-Preschool; ROC-AUC, area under the Receiver Operating Characteristic curve

## Discussion

The present study is the first to investigate the feasibility and validity of GT1M ActiGraph accelerometer measurements in a convenience sample of 1- to 2-year old toddlers. Furthermore, this study is also the first to examine if ActiGraph cut points developed among preschool children (3- to 5-year olds) are appropriate to accurately identify directly observed physical activity intensities in a toddler population.

To test the feasibility of the GT1M ActiGraph accelerometer, 47 toddlers from 11 child care centers wore the device during waking hours for 6 consecutive days. It is of concern that many day care centers were not interested in participating in the present study (response rate of 61.4%). A possible explanation could be that the contacted child care centers were anxious that the results of the present study would be used to evaluate the quality of the child care center. There was also a very low parental response rate in this study (20.2%). Perhaps parents were reluctant to participate because the study included an observation of their child. Another possibility is that child care staff did not motivate the parents to participate in the present study as no incentives were provided for both the child care center and the parents. Based on the experience from this study it can be suggested to use a more active recruitment approach to recruit parents (e.g., parent information session) or to provide incentives for parents and/or child care centers to achieve a higher response rate in this age group.

Using the 70/80 rule, a minimum registration time of 7.5 h during weekdays and 7.7 h during weekend days was determined. Applying this criterion, resulted in 29 excluded weekdays and 36 excluded weekend days, and 17 out of 47 toddlers (36.2%) failing to meet the inclusion criteria. Compared to previous research in preschoolers, applying the 70/80 rule and requiring 3 eligible days for inclusion, the proportion of ineligible data is higher in the present study [21,32]. In the study of Van Cauwenberghe et al. [21] applying these decision rules resulted in 97 excluded days and 40 out of 154 5-year old children (26.0%) providing non-eligible data and in the study of Verbestel et al. [32] this resulted in 48 out of 261 3- to 5-year old children (18.4%) providing non-eligible data. The rather high proportion of data not eligible for inclusion can probably be explained by the fact that wearing the accelerometer in the present study was a responsibility of both the parents and the child care staff. As child care staff were not instructed to fill in a diary, no information is available on the times the accelerometer was put on and taken off and the reasons for doing so when the child was at child care. One way to increase compliance could be to provide reminders (e.g., daily telephone calls) to parents and/or child care staff to put the accelerometers back on after sleeping and napping. Additionally, future studies could test the feasibility of wearing the accelerometer during day time napping as this would alleviate the issue of parents and child care staff having to remove and refit the monitors during the day. Further, it is possible that the inclusion criterion of 3 eligible days was too high in this population. If a criterion of 2 eligible days had been applied, another 8 toddlers (17.0%) could have been included for analyses. However, this suggestion could possibly decrease the reliability of the measurements as previous research in preschool-aged children indicated that the number of days of monitoring was more important to reliability than the number of hours [22]. Future research should establish the minimum number of days accelerometers need to be worn in order to represent habitual physical activity in toddlers. Ultimately, mean registration time of the included days in the present study was 9.4 h during weekdays and 9.9 h during weekend days. Considering the parental reported sleeping patterns of the toddlers, namely 11 h sleeping per night and 2 h napping per day, these findings suggest that there was good compliance to the study protocol. Further, a threshold of 7.5 hours registration time per day appears to be a reasonable suggestion for future research in this age group. Nevertheless, further research is required to determine the variability in toddlers' physical activity behavior within days and the minimum number of minutes required to represent a typical day in toddlers.

To evaluate the validity of the GT1M ActiGraph accelerometer, observations during free play at child care were conducted. Results of the free play observations were similar to previous research at child care in 2- to 3-year old children with the majority of the observations classified as stationary and motionless to stationary with movement of limbs or trunk behavior and a minority as moderate to fast movement [13]. The proportion of time spent in each activity level during free play was highly variable between toddlers, reflecting the different activities being undertaken by toddlers during free play. Descriptive statistics of the accelerometer output revealed that median ActiGraph activity counts increased in accordance with physical activity intensity but also demonstrated substantial variability. Results of a previous study, using a second-by-second coding protocol and the activity levels categories of the Children's Activity Rating Scale (CARS), indicate that mean accelerometer outputs for sedentary behavior, light physical activity, moderate physical activity and vigorous physical activity during free play are systematically higher in preschoolers, namely 448 ± 196, 734 ± 185, 823 ± 182 and 1115 ± 233 counts per 15 s, respectively [21]. Several explanations are possible for this discrepancy in accelerometer output during free play, including differences in the observation system, the protocol and the activities undertaken during free play and age related changes in anthropometrics, movement patterns and walking biomechanics.

In the present study, the criterion validity of the GT1M ActiGraph accelerometer for measuring physical activity in toddlers was considered acceptable (r = 0.66 and ρ = 0.52). A recent review, summarizing the evidence on the validity of the ActiGraph accelerometer to assess physical activity in older children and adolescents, suggests that the results of the present study are in line with previous validation studies where ActiGraph activity counts were moderately to highly correlated with observed activity (r = 0.52 - 0.77)[11].

Accelerometer activity counts are a dimensionless unit and researchers have attempted to calibrate these counts into biologically meaningful and interpretable data, such as time spent in activity levels of different intensities [10,18,19]. Calibration studies in toddlers are lacking, but numerous investigations involving preschool children have attempted to calibrate ActiGraph activity counts [10]. Therefore, the present study aimed to explore whether previously developed ActiGraph cut points for 3- to 5-year old children [21,23,25] allow for the accurate categorization of directly observed physical activity intensities in toddlers. It is critical to understand which cut points are able to accurately classify physical activity intensity in young children as others demonstrated that lack of consensus on this issue results in an inability to estimate population prevalence levels of physical activity in young children [21,33].

The Bland-Altman plots illustrate that the mean bias between directly observed and predicted time spent in each activity intensity was the lowest when the Pate cut points were used. Yet, wide limits of agreement were found, indicating that the time in the directly observed physical activity intensities was not accurately classified. Large mean differences and wide limits of agreement were established for the other two sets of cut points, suggesting that they were unable to accurately identify time spent in each observed activity intensity level in toddlers. To evaluate the predictive validity of the cut points thoroughly, sensitivity, specificity and ROC-AUC were calculated. The Pate sedentary behavior cut point performed fairly well to classify activity counts as stationary and motionless to stationary with movement of limbs or trunk behavior while the Sirard and Van Cauwenberghe sedentary behavior cut points exhibited an unacceptably high false positive rate. These findings do support the use of the Pate cut point to define sedentary time and non-sedentary time (a combination of light, moderate and vigorous physical activity) in toddlers. With respect to detecting slow, easy movement, all three cut points performed poorly. These findings endorse the development of toddler specific light physical activity cut points. Finally, for the purpose of categorizing activity counts as moderate to fast movement, all three moderate-to-vigorous physical activity cut points performed poorly as a function of a low true positive rate, indicating the need for toddler specific cut points to classify moderate-to-vigorous physical activity. Most importantly, the cut points currently used, appeared to be too high to accurately identify the time toddlers spent in moderate to fast movement. A very important consideration is that the GT1M ActiGraph is a hip-mounted accelerometer and measures accelerations in the vertical plane. Consequently, the accelerometer registers a reduced amount of vertical acceleration when non-ambulatory activities with limited trunk movement occur (e.g., climbing, pulling, pushing, peddling on a tricycle), resulting in misclassification of light physical activity as sedentary behavior or moderate-to-vigorous physical activity as light physical activity. Moreover, results from the present study illustrate that toddlers often engage in such activities during free play at child care. Therefore, combining accelerometers with monitors capable of detecting posture, using multiple monitors to measure movement of the trunk and the limbs simultaneously or applying pattern recognition may provide more accurate information beyond the capability of the GT1M ActiGraph when defining physical activity intensities in toddlers as well as in older children [10,17,18,34]. Research in these areas is urgently needed.

Some limitations of the present study need to be acknowledged. To measure physical activity intensity during free play, a 15 seconds measurement interval was used for both the accelerometers and the OSRAC-P. A 15 seconds measurement interval has been put forward to measure the spontaneous activities in young children [9,10]. However, there is a possibility that the 15 seconds measurement interval does not allow for the accurate detection of intermittent changes in physical activity intensity and fidgeting might be missed [10,35]. Furthermore, the OSRAC-P protocol requires to code the highest level of activity and the corresponding activity type during the 15 seconds observation interval which may mask other activity intensities and types during the observation interval. Especially for the classification of slow, easy movement and moderate to fast movement, it can be expected that levels of agreement were reduced because of this coding system. A continuous coding protocol may have been more appropriate to capture physical activity intensity and type during free play. Further, although the OSRAC-P decision rules to classify physical activity intensities in young children are well-established [7,26], the classification of standing as sedentary behavior is questionable [36]. Finally, the present study was limited by the small convenience sample used. Larger and more variable samples are needed to determine if individual factors, such as body size and motor development, modify the findings.

Several strengths of the present study are also noteworthy. First, the ActiGraph accelerometer was validated against directly observed free play activities with excellent inter-observer reliability. Additionally, by using direct observation, in-depth information on the physical activity types of toddlers was gathered. Second, the criterion validity and the predictive validity were evaluated using appropriate statistical approaches [7,10].

## Conclusions

In summary, the results of the present study endorse the use of the GT1M ActiGraph accelerometer to assess habitual physical activity in free-living toddlers. In addition, the present findings suggest that the Pate cut point can be used to classify sedentary behavior and non-sedentary behavior in toddlers. However, further research is warranted to classify sedentary time in toddlers with excellent accuracy using accelerometry. None of the three cut points developed among preschool children appeared to be suitable to differentiate light and moderate-to-vigorous physical activity in this age group. Until such equations are developed in toddlers, researchers could use accelerometer counts (e.g., counts per minute) as a measure of physical activity participation in toddlers.

## List of abbreviations

OSRAC-P: Observational System for Recording Physical Activity in Children-Preschool; ROC-AUC: area under the Receiver Operating Characteristic curve.

## Competing interests

The authors declare that they have no competing interests.

## Authors' contributions

All authors made substantial contribution to the design of the study. EVC collected the data. EVC and JG conducted the data processing. EVC analyzed and interpreted the data and drafted the manuscript. All authors contributed to the writing of the manuscript and have read and approved the final version.
